# Building global mental health research capacity: the collaborative hubs for international research on mental health

**DOI:** 10.1017/gmh.2018.24

**Published:** 2018-10-23

**Authors:** LeShawndra N. Price

**Affiliations:** National Institute of Mental Health, National Institutes of Health, Bethesda, MD, USA

**Keywords:** Career development, global mental health, research capacity-building, research training, workforce development

This special collection of articles from *Global Mental Health* features papers on research training and capacity-building efforts in low- and middle-income countries originally supported through the Collaborative Hubs for International Research on Mental Health (CHIRMH), an initiative of the National Institute of Mental Health (NIMH) at the National Institutes of Health (USA). First released in 2010, the CHIRMH funding opportunity announcements†^1^ stimulated research and research capacity-building to increase the evidence base for mental health interventions in World Bank designated low- and middle-income countries (LMIC). Five grants were awarded through the CHIRMH program.^2^ Each funded research award conducted global mental health research addressing the mental health treatment gap, the proportion of persons who need, but do not receive, care; and provided capacity-building opportunities regionally. This special collection of articles reflects the research capacity-building focus of the CHIRMH initiative.

In 2010, mental and substance use disorders were the leading cause of years lived with disability worldwide (Whiteford *et al*., 2013) and global financial resources for mental health in LMICs remained low compared with high-income countries (WHO, 2011). In consideration of trends in incidence and prevalence of mental disorders globally and variations in access to care across populations and countries, that same year, the National Institutes of Health convened scientific experts, thought leaders, and global mental health stakeholders (e.g. service providers, representatives of mental health service users, policymakers, and non-governmental organizations) from LMICs to identify strategic research opportunities that the NIMH could prioritize to increase global mental health research on the prevention and cure of mental illnesses, thereby leading to improved mental health worldwide. Specifically, the workshop aimed to: identify areas of research that would enable community mental health service providers in LMICs, policymakers, advocates, researchers, and their partners to advance treatment and rehabilitation of mental illness; identify areas of need and close gaps in implementation research; and articulate the ways policymakers, advocates, researchers, service providers, and others could partner to facilitate research that informs implementation and scale-up of evidence-based interventions. Three key themes emerged from the workshop: (1) a need for research on mental health whose findings could be translated for use by policymakers (e.g. research findings provide evidence for investment in mental health services); (2) need for more mental health service providers in the face of inadequate numbers in most LMICs; and (3) development, implementation, and scale-up of mental interventions tailored to match local contexts and resources. Undergirding each of these themes was the need for a diverse scientific workforce capable of conducting research to address these thematic areas. In response to key recommendations from the workshop, the NIMH included a focus on research capacity building within the CHIRM funding opportunity announcement.

Four of the papers within this special collection describe specific capacity efforts in three geographic regions (i.e. Southeast Asian, Sub-Saharan Africa, Latin-America) amongst four of the funded grants. A fifth paper describes how the capacity building was monitored and evaluated across all five awarded grants. Collectively, the articles in this collection speak to the diversity of strategies employed to train and develop researchers in global mental health in LMICs. These articles also highlight successful capacity-building efforts tailored to local contexts within geographic regions and within specific countries.

Sharma & Razzaque (2017) present a multi-faceted capacity building strategy used in Southeast Asia for building research capacity through distance learning and mentoring, yearly short courses on topics relevant to the region, as well as traditional mentoring and research apprenticeship. The article presents a comprehensive approach for both building individual capacity and strengthening institutional research infrastructure. The article also discusses the development and implementation of an online mentoring platform, which was unique amongst the CHIRMH awardees.

The article by Yang *et al*. (2017) describes a partnership between investigators in the USA and throughout Latin America to develop a regional research capacity-building network. Key to the success of the network in building research capacity was first training early career researchers on the precipice of research independence who then subsequently trained additional early career investigators. The article uses case studies to illustrate the success of the research capacity-building strategies used in building capacity of individuals and a self-sustained network within Latin America. Building upon existing research capacity-building frameworks, Bonini *et al*. (2017) also describe research capacity-building within Latin America, which was tailored to needs within the country and within the region.

Schneider *et al*. (2016*a*) describe successes in building research capacity in Sub-Saharan Africa, including training more than 100 individuals in six countries, and challenges, such as ensuring long-term sustainability. In addition, the article describes the launch of an M. Phil. Program in Public Mental Health, which focused on equipping full-time mental health practitioners with basic research skills.

The final paper in the collection by Schneider *et al*. (2016*b*) reviews capacity building metrics across regions, LMICs, grant awards, and the capacity-building activities supported by the CHIRMH. In addition, the article details inter- and intra-hub capacity-building activities, monitoring and evaluation approaches across the CHIRMH, and short- and medium-term capacity-building outcomes. Challenges are also discussed.

The articles in this collection provide examples of contextually relevant approaches and strategies for building individual, institutional, and regional research capacity in LMICs. The work synthesized in this special collection provides a unique insight into the challenges, advances, successes, and early wins experienced by those committed to systematically building research capacity for mental health services and implementation research globally. In the future, short-, medium-, and long-term strategies will be needed that expand efforts developed through the CHIRMH initiative. In addition, new efforts will be needed to address persistent challenges and newly identified gaps. The articles not only serve as a model for what can be done when resources are mobilized, and focused training and career development opportunities are offered, but also as a catalyst for stimulating continued efforts in this area. The collection also highlights the need for continued investment in building the next generation of researchers that will be prepared to conduct policy-relevant research to improve the mental health treatment gap and effective delivery of mental health services. Doing so will have a transformative impact, ultimately leading to improved mental health care worldwide.

## Footnotes

1 Awards funded through the Collaborative Hubs for International Research on Mental Health (CHIRMH) initiative responded to one of two funding opportunity announcements, RFA-MH-11-070 or RFA-MH-12-110.

2 Descriptions of the five awards funded through the CHIRMH initiative can be found at https://www.nimh.nih.gov/about/organizaion/gmh/globalhubs/index.shtml.
